# Cytotoxicity evaluation of dental and orthodontic light‐cured composite resins

**DOI:** 10.1002/cre2.337

**Published:** 2020-10-25

**Authors:** Raoul Bationo, Ablassé Rouamba, Abdoulaziz Diarra, Monique Lydie Ahia Beugré‐Kouassi, Jean‐Bertin Beugré, Fabienne Jordana

**Affiliations:** ^1^ CHU de Bogodogo Ouagadougou Burkina Faso; ^2^ Université Félix Houphouët‐Boigny Abidjan Côte d'Ivoire; ^3^ Laboratoire de Biochimie et Chimie Appliquées Université Joseph Ki‐Zerbo Ouagadougou Burkina Faso; ^4^ CHU de Tengandogo/UFR Sciences de la Santé Université Joseph Ki‐Zerbo Ouagadougou Burkina Faso; ^5^ Département d'Orthopédie Dento‐Faciale – UFR d'Odonto‐Stomatologie Abidjan Côte d'Ivoire; ^6^ Service d'Odontologie – CHU Nantes UFR d'Odontologie de Nantes Nantes France

**Keywords:** cytotoxicity, GC–MS, light‐cured composite resin, monomers

## Abstract

**Introduction:**

The aim of this study was to determine the cytotoxicity of light‐cured composite resins (Clearfil ES‐2, Clearfil ES Flow, Filtek Supreme XTE, Grengloo, Blugloo, Transbond XT, and Transbond LR) then to assess leachable components in contact with human gingival fibroblasts (GFs) and to quantity detected bisphenol A (BPA).

**Methods:**

Light‐cured composite resin discs were immersed for 24 hours in gingival fibroblastic medium (*n* = 3 for each product) and in control medium (*n* = 2 for each product) contained in plate. Cytotoxicity of the products (*n* = 95) was determined by the measure of cell viability using MTT assay after reading the optical densities of the plates. The analysis of leachable components was done by gas phase chromatography and mass spectrometry (GC–MS) and detected BPA was quantified. The limit of quantification was 0.01 μg/mL. Statistical analyses were performed by using IBM SPSS Statistics 20 and Kruskal–Wallis and Mann–Whitney *U*‐tests were applied.

**Results:**

Cell viabilities were between 85 and 90%. Many chemical compounds including triethylene glycol dimethacrylate (TEGDMA) and BPA were identified. The average concentrations were 0.67 μg/mL ± 0.84 in the control medium and 0.73 μg/mL ± 1.05 in the fibroblastic medium. Filtek Supreme XTE presented the highest concentration of BPA with 2.16 μg/mL ± 0.65 and Clearfil ES Flow presented the lowest with 0.25 μg/mL ± 0.35. No BPA was detected with Transbond XT and Transbond LR. Clearfil ES Flow, Filtek Supreme XTE, Grengloo and Transbond LR presented residual TEGDMA.

**Conclusions:**

Light‐cured composite resins are slightly cytotoxic opposite GFs and release many components including BPA and TEGDMA. Clinical precautions should be taken to decrease the release of these monomers.

## 1 INTRODUCTION

Bisphenol A (BPA) based dental resins are commonly used in preventive and reparative dentistry and in orthodontics. The composite resins used in dentistry are complex polymers containing a variety of monomers, initiators, activators, stabilizers, plasticizers and other additives. Two monomers are mainly used: bisphenol A diglycidyl dimethacrylate (Bis‐GMA) and triethylene glycol dimethacrylate (TEGDMA) (Bationo et al., 2019).

BPA is never present in pure state; it is used as raw material for the formulation of Bis‐GMA (Perez‐Mondragon et al., 2020).

In recent years, the increasing presence of polymers in oral cavity has raised questions about safety of resin matrix components. Despite their increasing popularity, it is worrying that composite resins can be toxic due to the fact that they can release components (Reichl et al., 2008).

The toxicity of resin‐based materials is due to residual monomers as well as to degradation products linked to activity of the salivary esterases. Elution of BPA may result from impurities left after synthesis of resin due to incomplete polymerization or degradation of this resin (Schmalz, Preiss, & Arenholt‐Bindslev, 1999; Van Landuyt et al., 2011).

The literature review confirms toxicity of BPA. BPA is an endocrine disruptor with potential toxicity in vitro (Wetherill et al., 2007) and in vivo (Richter et al., 2007).

Other compounds including TEGDMA and Bis‐GMA (Al‐Hiyasat, Darmani, & Elbetieha, 2002; Al‐Hiyasat, Darmani, & Elbetieha, 2004; Gioka et al., 2009; Kloukos, Pandis, & Eliades, 2013; Nathanson, Lertpitayakun, Lamkin, Edalatpour, & Chou, 1997; Volk, Leyhausen, & Geurtsen, 2012; Wada, Tarumi, Imazato, Narimatsu, & Ebisu, 2004; Wisniewska‐Jarosinska et al., 2011), released by restorative and bonding composites, also present potential toxicity. Infants, young children and pregnant or lactating women are the most sensitive (Shelby, 2008).

The release of these components into the surrounding tissue may cause adverse local reaction or even systemic effects (Lönnroth & Shahnavaz, 1997; Mathias, Caldwell, & Maibach, 1979; Schmalz, 1998).

Composite resins are extensively used as restorative materials because of esthetic demands and concerns over adverse effects of mercury from amalgam (Bakopoulou, Triviai, Tsiftsoglou, & Garefis, 2006). In orthodontics, composite resins are the materials of choice to bond orthodontic accessories to dental enamel (Bishara et al., 2007; Paschos et al., 2009). Regarding dental treatments, it is advantageous to maintain maximal tissue vitality and cytotoxic reactions must be prevented, which necessitates the dental compounds to be screened before they are used clinically (Murray, Godoy, & Godoy, 2007).

Dental and orthodontic light‐cured composite resins whose composition includes BPA derivatives and TEGDMA are studied. The aim of this study was to determine the cytotoxicity of light‐cured composite resins when in contact with human gingival fibroblasts (GFs), to assess leachable components and to quantity detected BPA in the fibroblastic medium and the control medium.

## 2 MATERIALS AND METHODS

### 2.1 Preparation of resin discs

Discs 10 mm in diameter and 1 mm thick were prepared from dental and orthodontic light‐cured composite resins (Table 1). The discs were cured at the top surface for 20 seconds using BA Optima 10 LED Curing Light (light intensity 1,000–1,200 mW/cm^2^ and wavelength 420–480 nm).

**TABLE 1:** Characteristics of resins used in the study

Manufacturer	Product (lot)	Resin matrix
Kuraray	Clearfil majesty ES‐2 (4D0069)	Bis‐GMA, hydrophobic aromatic dimethacrylate hydrophobic aliphatic dimethacrylate
	Clearfil majesty ES flow (A60239)	TEGDMA, hydrophobic aromatic dimethacrylate
Ormco	Grengloo (6623923)	TEGDMA, UDMA, HEMA, Bis‐EMA6, GMA, EO‐TMPTA, 3‐trimethoxysilylpropyl methacrylate
	Blugloo (6556174)	UDMA, Bis‐EMA6, GMA, EO‐TMPTA, 3‐trimethoxysilylpropyl methacrylate
3 M	Transbond XT (N921496)	Bis‐GMA, Bis‐MEPP
	Transbond LR (N919866)	Bis‐GMA, TEGDMA
	Filtek supreme XTE (N879475)	Bis‐GMA, UDMA, TEGDMA, Bis‐EMA6, PEGDMA

### 2.2 Cytotoxicity testing

Fibroblasts were cultured from gum operative waste obtained after dental extraction in a patient who signed a consent form. The explants are rinsed with phosphate buffered saline and then laid onto the connective side in a 100 mm^2^ Petri dish. The dish is kept semi‐open under laminar flow for 30 minutes so that explants can adhere to the culture surface. RPMI 1640 culture medium is instilled on the explants to prevent them from drying out and to maintain cell viability.

About 10 mL of RPMI 1640 culture medium supplemented with fetal bovine serum 10%, Penicillin (10,000 U/mL)/Streptomycin (10 mg/mL) 1% and l‐Glutamine (200 mM) 1%, is poured in the dish then it is stored in humidified incubator at 37°C under 5% CO_2_ in air.

The culture medium is replaced every two days up to confluence. Cells are then trypsinized (Trypsin 0.25% in EDTA 0.02%) for 3 minutes at 37°C.

Each resin disc was immersed in 400 μL (ISO 10993‐12:2012 [ISO, 2012] standard for medical‐device testing in biologic systems) of fibroblastic medium (*n* = 3 for each product) and control medium (*n* = 2 for each product) contained in a 12‐well plate and kept for 24 hours in humidified incubator at 37°C under 5% CO_2_ in air. 100 μL of fibroblastic medium (*n* = 95) were then injected onto 96‐well plates.

About 10 μL (5 mg/mL) of MTT solution were added to each well (96‐well plates) and plates were incubated for 3 hours. MTT solution was then removed and 100 μL of dimethyl sulphoxide were added to each well. The plates were shaken and then optical densities (OD) were measured in a plate reader (EPOCH) at a wavelength of 570 nm. The fibroblastic medium was used as cell control (*n* = 7). Cell viability was calculated using the formula (Vande Vannet, Mohebbian, & Wehrbein, 2006): Cell viability = (OD test group/OD cell control) × 100.

### 2.3 Gas phase chromatography and mass spectrometry analysis

The eluates of incubation solutions (fibroblastic medium and control medium) were extracted using solid phase extraction (NH_2_ cartridge) and then analyzed by gas phase chromatography and mass spectrometry (GC–MS) (Agilent 6890 Series – Agilent 7673). The control medium was a pure cell culture medium; samples immersed in this medium were used as control group. A capillary column 30 m in length, internal diameter of 320 μm and film thickness of 0.25 μm was used with helium carrier gas at a flow rate of 1.2 mL per minute. The column temperature program was set as follows: initially, 80°C for 1 minute, increasing to 150°C at a rate of 20°C per minute and then increasing to 280°C for 2 minutes at a rate of 10°C per minute. The injector temperature was 280°C and the transfer line was 280°C. Mass spectra were obtained using electron impact ionization (69.9 eV, 34.6 μA, and 230°C).

Data were acquired by scan mode and selected ion monitoring (SIM) mode and were processed with MSD ChemStation software.

The presence of fragments of BPA (91‐119‐213‐228) and TEGDMA (41‐69‐86‐113) in SIM mode allows the identification of these compounds.

BPA calibration curve and response factor were computed with reference BPA and caffeine as internal standard. Linear correlation with efficiency of 0.996 was obtained between BPA amount and corresponding peak area. BPA was quantified after his identification. The limit of quantification was 0.01 μg/mL.

### 2.4 Statistical analysis

Statistical analyses were performed by using IBM SPSS Statistics 20 and means and standard deviations were calculated for descriptive statistical analysis. Because the data did not show a normal distribution, a significant difference was evaluated using the Kruskal–Wallis test and the Mann–Whitney *U‐*test at a significance level of *p* < .05.

## 3 RESULTS

### 3.1 Cytotoxicity

The average values of OD of each group (tested composite resins and cell control) were presented in Table 2. The highest OD were respectively those of cell control (0.637) and Transbond LR (0.573). The lowest OD was observed with Blugloo (0.543). There was significant difference between the studied groups (*p* < .05).

**TABLE 2:** Average values of optical densities

Groups (*n* = 102)	Optical density (SD)	*p* Value
Clearfil ES‐2 (*n* = 14)	0.564 (0.03)	.005*
Clearfil ES flow (*n* = 14)	0.564 (0.05)	
Filtek supreme XTE (*n* = 12)	0.568 (0.03)	
Grengloo (*n* = 14)	0.565 (0.03)	
Blugloo (*n* = 14)	0.543 (0.04)	
Transbond XT (*n* = 14)	0.545 (0.03)	
Transbond LR (*n* = 13)	0.573 (0.04)	
Cell control (*n* = 7)	0.637 (0.02)	

Abbreviation: SD, standard deviation.

*p < .05.

**FIGURE 1:**
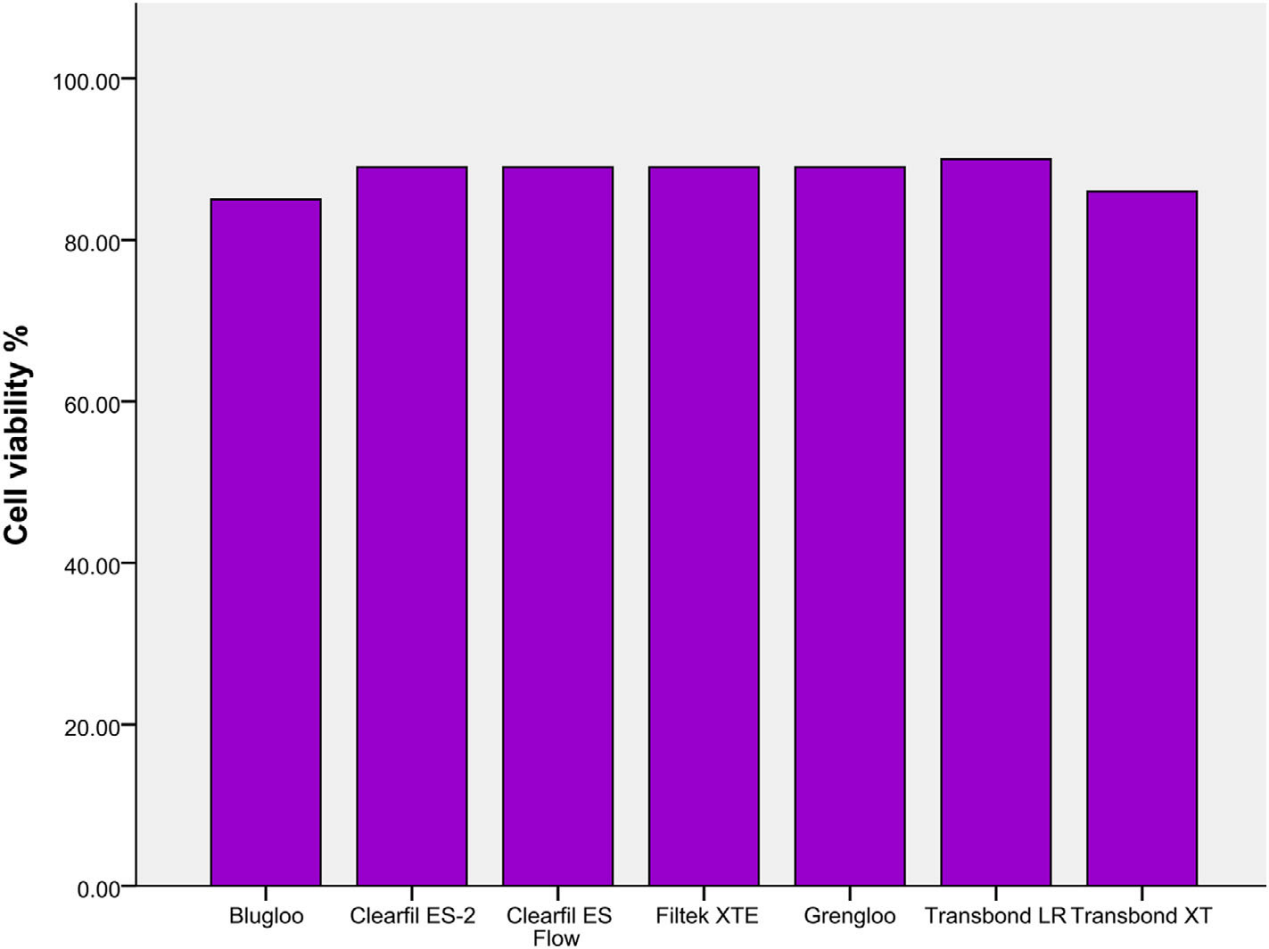
Cell viability

Figure 1 shows the cell viability of the composite resins. The cell viability of Transbond LR, Transbond XT and Blugloo were respectively 90%, 86% and 85% of cell. The other tested resins tested had all a cell viability of 89%.

### 3.2 GC–MS analysis

Many chemical compounds were identified in analyzed resin materials by GC–MS (Table 3) including BPA and TEGDMA.

**TABLE 3:** Compounds found

Compound	Clearfil ES‐2	Clearfil ES flow	Filtek supreme XTE	Grengloo	Blugloo	Transbond XT	Transbond LR
Hexadecenoic acid C_16_H_32_O_2_	X	X	X	X	X	X	X
Procaine C_13_H_20_N_2_O_2_	X	X	X	X	X	X	X
Bisphenol A C_15_H_16_O_2_	X	X	X	X	X		
Octadecanoic acid C_18_H_36_O_2_	X	X	–	X	–	X	
C_16_H_30_O_2_	X	X	–	–	X	–	X
TEGDMA C_14_H_22_O_6_	–	X	X	X	–	–	X
Methylparaben C_8_H_8_O_3_	–	–	–	X	X	–	X
C_20_H_38_O_2_	X	X	–	–	–	–	–
C_18_H_34_O_2_	–	X	–	X	–	–	
C_20_H_36_O_2_	–	X	–	–	–	–	X
*N*,*N*′‐Methylene bis acrylamide C_7_H_10_N_2_O_2_	–	–	–	X	–	X	–
C_18_H_32_O_2_	X	–	–	–	–	–	–
C_6_H_14_O_4_	–	–	–	–	X	–	–
EGDMA C_10_H_14_O_4_	–	–	–	X	–	–	–
*N*‐Octadecane C_18_H_38_	–	–	–	–	–	X	–
C_22_H_46_	–	–	–	–	–	X	–
C_8_H_9_NO	–	–	–	–	–	–	X
*N*,*N*‐Dimethyl benzocaine C_11_H_15_NO_2_	–	–	–	–	–	–	X

Table 4 shows the concentration of detected BPA. The average concentrations were 0.67 μg/mL ± 0.84 and 0.73 μg/mL ± 1.05, respectively in the control medium and in the fibroblastic medium. The highest concentration of BPA was observed with Filtek Supreme XTE (2.16 μg/mL ± 0.65) and the lowest concentration was 0.25 μg/mL ± 0.35 with Clearfil ES Flow. No BPA was detected with Transbond XT and Transbond LR. There was not an impact of the medium on the concentration of released BPA (*p* = .7) but there was a significant difference between the average concentrations of BPA released from each product (*p* < .001).

**TABLE 4:** Concentration of BPA detected (μg/mL)

Characteristics		Average concentration (SD)	*p* Value
Medium (*n* = 35)			.7
	Control (*n* = 14)	0.67 (0.84)	
	Fibroblastic (*n* = 21)	0.73 (1.05)	
Products (*n* = 35)			<0.001*
	Clearfil ES‐2 (*n* = 5)	0.46 (0.44)	
	Clearfil ES flow (*n* = 5)	0.25 (0.35)	
	Filtek supreme XTE (*n* = 5)	2.16 (0.65)	
	Grengloo (*n* = 5)	0.34 (0.55)	
	Blugloo (*n* = 5)	1.75 (1.02)	
	Transbond XT (*n* = 5)	0.00 (0.00)	
	Transbond LR (*n* = 5)	0.00 (0.00)	

Abbreviation: SD, standard deviation.

*p < .05.

**FIGURE 2:**
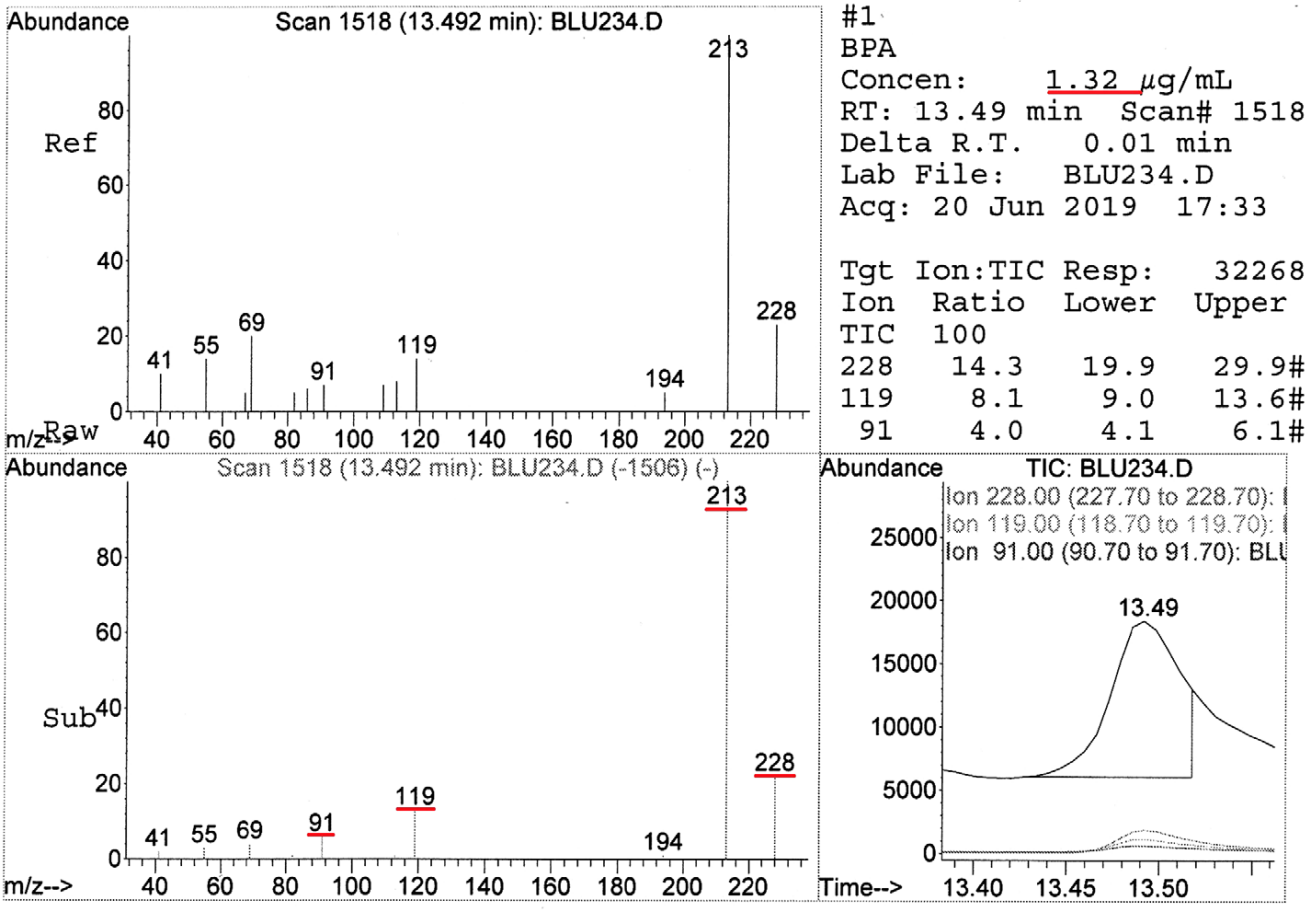
Spectrum and concentration of BPA detected with Blugloo immersed in the fibroblastic medium. BPA, bisphenol A

Figure 2 shows a concentration of BPA detected (1.32 μg/mL) and the abundance of the fragments (91‐119‐213‐228) after immersion of Blugloo in the fibroblastic medium.

Clearfil ES Flow, Filtek Supreme XTE, Grengloo and Transbond LR presented residual TEGDMA with the two‐immersion media.

**FIGURE 3:**
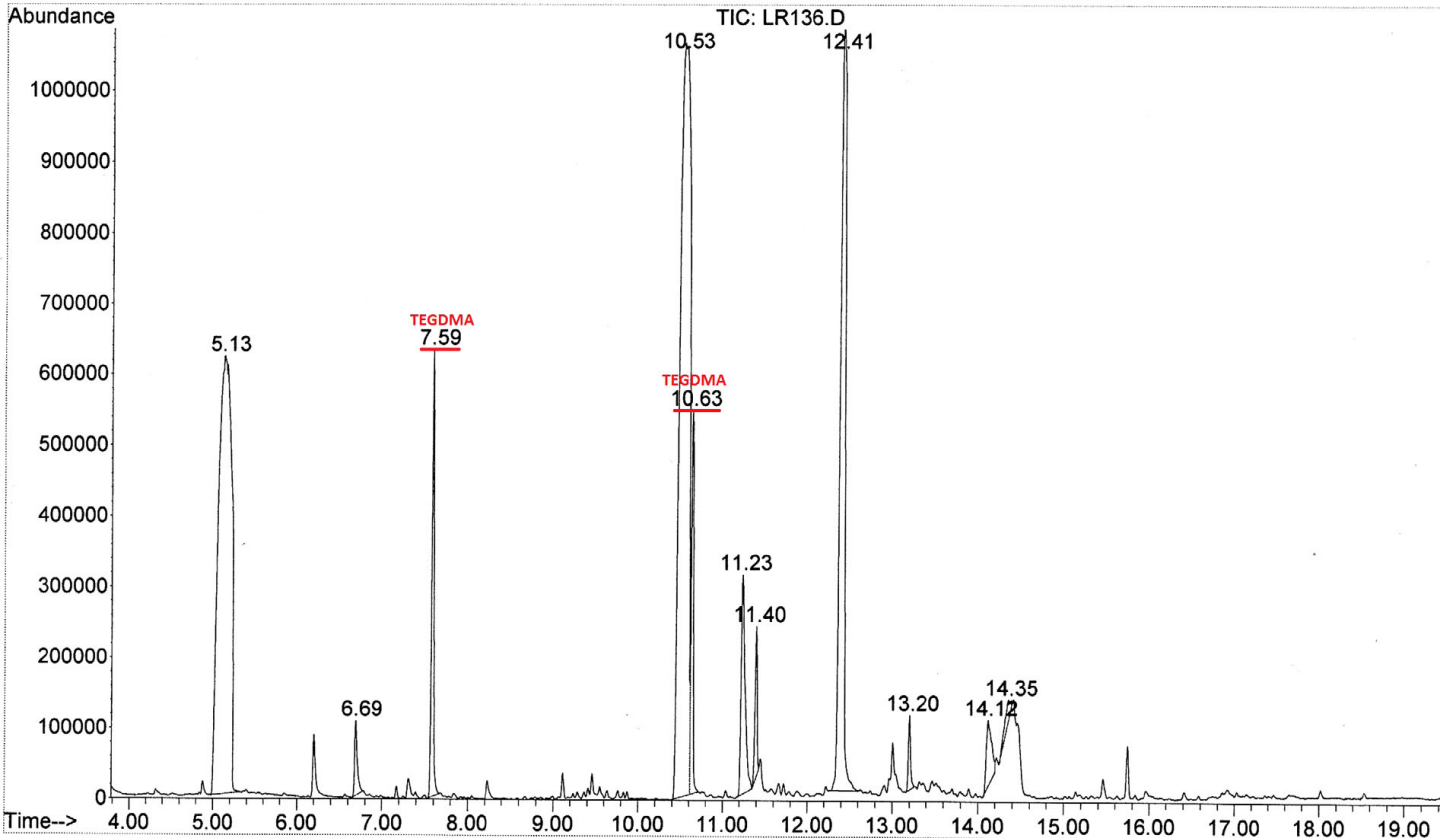
Mass spectrum showing TEGDMA detected with Transbond LR immersed in the fibroblastic medium. TEGDMA, triethylene glycol dimethacrylate

Figure 3 shows a mass spectrum of Transbond LR immersed in the fibroblastic medium and indicating the presence of TEGDMA.

## 4 DISCUSSION

Transbond LR was the most cytotoxic materials with more closer results to Clearfil ES‐2, Clearfil ES Flow, Filtek Supreme XTE and Grengloo. Blugloo and Transbond XT remained the least cytotoxic, when compared with the other materials. According to the method of Sjogren, Sletten, and Dahl (2000), all resin‐based materials of this study are slightly cytotoxic (cell viability between 60 and 90%).

Regarding cell viability, our results match with those of Pudpong, Anuwongnukroh, Dechkunakorn, Wichai, and Tua‐ngam (2018) where Transbond XT and Grengloo had cytotoxic potential during the first day and Grengloo had the highest cell viability compared to Transbond XT.

A review of genotoxicity and cytotoxicity of orthodontic bonding adhesives by Riaz, Norma, and Thirumulu (2019) noted that Transbond XT was the mostly used material in studies and there were some differences in the result regarding the cytotoxicity of Transbond XT in different studies. Two research groups found it to be non‐cytotoxic (Ahrari, Tavakkol Afshari, Poosti, & Brook, 2010; Angiero et al., 2009) and one group mentioned it as less cytotoxic compared to the dual cured orthodontic adhesives (Jagdish et al., 2009).

GFs are used for cytotoxicity testing because they are close to dental restorative materials in oral cavity and are more clinically relevant. GFs are also sensitive cells that can be easily isolated and grown in normal culture medium (Hensten‐Pettersen & Helgeland, 1981).

It has been shown that monomer release from composite resins is completed within 24 hours whence the 24‐hours exposure time of GF to composite resins (Ferracane & Condon, 1990). Therefore, most of toxic effects of composite resins occur within first 24 hours.

The polymerization and conversion of monomer in organic matrix of resin‐based adhesive materials is rarely complete and this seems to be responsible for most of reported adverse effects such as cytotoxicity, allergy and inflammatory potential (Borelli et al., 2017; Goldberg, 2008).

The rate of polymerization can significantly affect cytotoxicity of composite material by diffusion of a large number of unpolymerized resin monomers ( Ferracane, 1994; Ferracane & Condon, 1990; Geurtsen, Lehmann, Spahl, & Leyhausen, 1998). Several monomers contained in composite resins (such as Bis‐GMA, Bis‐EMA, UDMA, TEGDMA declared by manufacturer) are known to diffuse from partially polymerized materials and to be cytotoxic in vitro (Geurtsen et al., 1998; Hanks, Strawn, Wataha, & Craig, 1991).

Several studies show that dental adhesives are cytotoxic for GF (Huang, Tsai, Chen, & Kao, 2002; Szep, Kunkel, Ronge, & Heidemann, 2002). It is mainly the residual adhesive monomers that cause gingival inflammation and irritation (Gioka et al., 2005). To avoid undesirable side effects of adhesives on gingival tissue, it is preferable to polymerize it quickly after it has been applied (van Gastel, Quirynen, Teughels, Coucke, & Carels, 2007).

Malkoc, Corekci, Ulker, Yalcin, and Sengun (2010) found significant similarities in resin matrix of five different photopolymerizable orthodontic composites when assessing the ingredient of tested materials. However, Transbond XT contained Bis‐EMA. Bis‐EMA monomer showed analogous cytotoxic effect to that of TEGDMA (Geurtsen et al., 1998). Transbond XT cytotoxicity could therefore be explained by the presence of Bis‐EMA in its matrix. The mechanism of cytotoxicity induced by TEGDMA on human fibroblasts has been studied by Stanislawski et al. (2003).

Schubert, Ziegler, Bernhard, Burgers, and Miosge (2019) concluded that Ormocer (Admira Fusion) has superior biocompatibility in vitro compared to dimethacrylate‐based composites (GrandioSo and Filtek Supreme XTE).

TEGDMA and HEMA monomers are cytotoxic towards gingival cells and are probably responsible for allergies to these materials. In addition, unbound monomers promote bacterial growth particularly the microorganisms involved in dental caries formation (Goldberg, 2008).

The addition of hydrogen peroxide (whitening component) even at low doses, potentiates toxicity of TEGDMA and UDMA opposite human gingival and pulp fibroblasts but remains without effect on Bis‐GMA and HEMA (Reichl et al., 2008).

Even completely light‐cured, HEMA is not fully linked; a part could be released and therefore an allergic reaction is possible. HEMA has been shown to be able to pass through the dentin tubules and end up in pulp tissue. Several cases of allergic reactions are reported in literature (Bryant, 2016).

TEGDMA, Bis‐GMA and UDMA have toxic effects on GF and HaCaT cells. Bis‐GMA is the most toxic and UDMA the least toxic (Moharamzadeh, Van Noort, Brook, & Scutt, 2007).

The potential toxicity of various associated monomers seems to be greater than toxicity of each monomer studied individually. Cytotoxicity on GF is not the same depending on monomer and can be hierarchized as follows: HEMA<TEGDMA<UDMA<Bis‐GMA (Reichl et al., 2006).

Eluted monomers from composite resins are of great clinical importance because of their cytotoxic effects on GF and macrophages (Issa, Watts, Brunton, Waters, & Duxbury, 2004; Michelsen et al., 2012; Moharamzadeh et al., 2007).

In most studies, cytotoxicity ranking of basic monomers is as follows: Bis‐GMA>UDMA>TEGDMA>HEMA (Darmani, Al‐Hiyasat, & Milhem, 2007; Issa et al., 2004; Moharamzadeh et al., 2007).

In the present study, all the resins that released BPA contained BPA derivatives in their composition except Clearfil ES Flow. The BPA detected in Clearfil ES Flow could be due to contamination or either the manufacturer has not mentioned all the ingredients in the safety data sheet. The comparison of the average concentrations of BPA released in the fibroblastic and control media indicated that the medium has no impact on the concentration of BPA. The resins containing TEGDMA in their composition released this compound.

Regarding BPA, current results match with those of Bationo, Jordana, Boileau, and Colat‐Parros (2016) who detected BPA from Blugloo and not from Transbond XT after 24 hours of immersion in Milli‐Q water.

In a study of Polydorou, König, Hellwig, and Kümmerer (2009) including an Ormocer, Bis‐GMA was detected in greater quantities than TEGDMA in Filtek Supreme XT and Ceram X. BPA was found in non‐polymerized Filtek Supreme XT and Ceram X eluates and in polymerized Ceram X immersion eluates.

BPA elution could result from impurities left after resin synthesis, first due to incomplete polymerization, and later due to degradation of resins (Schmalz et al., 1999; Van Landuyt et al., 2011).

Studies on BPA have focused on hormonal activity (Chao et al., 2012). BPA concentrations >0.01 mmol/L are noted to have effect on estrogen (Kita et al., 2009).

Komurcuoglu, Olmez, and Vural (2005) and Polydorou, Trittler, and Hellwig (2007) reported higher amount of eluted Bis‐GMA compared to other monomers in their studies. This was explained by the fact that the conversion of Bis‐GMA double bond is lower compared to other monomers (Stansbury & Dickens, 2001).

Bis‐GMA concentrations decrease cell viability in time and dose dependent (Cohn‐Inostroza, Ehrenfeld Slater, Pavicic Rojas, & De la Rosa Varela, 2015).

Article by Pulgar et al. (2000) mentioned release of Bis‐GMA, BADGE and BPA from various polymerized composites. The highest amounts of BPA are observed after 24‐hour immersion in water at pH 7.

High Performance Liquid Chromatography (HPLC) analysis identified release of bisphenol A glycerolate dimethacrylate, TEGDMA and diurethane dimethacrylate from composite resins (Tetric Evo Ceram, Tetric Ceram, Dyract Xtra, Filtek Supreme XTE, Admira) and shown that monomers release increases between 1 hour and 1 day but remains low (Frese et al., 2018).

Uomo et al. (2017) determined effect of orthodontic resins (Eagle Spectrum, Grengloo and Transbond XT) on cell viability by Alamar Blue test and evaluated released monomer both before and after resin polymerization using HPLC. The results showed the role of polymerized resin in determining cytotoxic effect of orthodontic resins and suggested that differences in chemical composition of resin matrix appeared to be much more related to decrease in cell viability than amount of monomer released from orthodontic resins.

## 5 CONCLUSION

Light‐cured composite resins are slightly cytotoxic opposite GFs and release many components including BPA and TEGDMA. Clinical precautions should be taken to decrease the release of these monomers.

Resin‐based materials that are used in dentistry should be harmless to oral tissues, so they should not contain any leachable toxic and diffusible substances that can cause some side effects.

## CONFLICT OF INTEREST

The authors declare no potential conflicts of interest.

## Data Availability

Data available on request from the author
